# Paratuberculosis in Captive Scimitar-Horned Oryxes (*Oryx dammah*)

**DOI:** 10.3390/ani10111949

**Published:** 2020-10-23

**Authors:** Claudio Pigoli, Chiara Garbarino, Matteo Ricchi, Eleonora Bonacina, Lucia Gibelli, Valeria Grieco, Erika Scaltriti, Paola Roccabianca, Giuseppe Sironi, Simone Russo, Stefano Pongolini, Norma Arrigoni

**Affiliations:** 1 Department of Veterinary Medicine (DIMEVET), University of Milan, Via dell’Università 6, 26900 Lodi, Italy; claudio.pigoli@unimi.it (C.P.); paola.roccabianca@unimi.it (P.R.); giuseppe.sironi@unimi.it (G.S.); 2 National Reference Centre for Paratuberculosis, Sede Territoriale di Piacenza, Istituto Zooprofilattico Sperimentale della Lombardia e dell’Emilia Romagna (IZSLER), Strada Faggiola 1, 29027 Gariga di Podenzano, Italy; chiaraanna.garbarino@izsler.it (C.G.); matteo.ricchi@izsler.it (M.R.); simone.russo@izsler.it (S.R.); norma.arrigoni@izsler.it (N.A.); 3 Parco Faunistico Le Cornelle, Via Cornelle 16, 24030 Valbrembo, Italy; eleonorabonacina@libero.it; 4 Histology Laboratory, Sede Territoriale di Milano, Istituto Zooprofilattico Sperimentale della Lombardia e dell’Emilia Romagna (IZSLER), Via Giovanni Celoria 12, 20133 Milano, Italy; luciarita.gibelli@izsler.it; 5 Risk Analysis and Genomic Epidemiology Unit, Istituto Zooprofilattico Sperimentale della Lombardia e dell’Emilia Romagna (IZSLER), Via dei Mercati 13/A, 43126 Parma, Italy; erika.scaltriti@izsler.it (E.S.); stefano.pongolini@izsler.it (S.P.)

**Keywords:** paratuberculosis, pathology, scimitar-horned oryx, whole-genome analysis

## Abstract

**Simple Summary:**

Paratuberculosis is a bacterial infection occurring globally in ruminants, with an impact on animal health and welfare. The chronic nature of the disease, the variability in infection progression, and immune response can make diagnosis difficult. The disease can have severe consequences in zoological parks where threatened animal species are hosted. In the present study, we investigated paratuberculosis in a group of scimitar-horned oryx, an endangered ruminant species, hosted in a Northern Italy zoological park. The animals were derived from a flock imported from Slovakia. We report the results of different diagnostic techniques and underline the contribution of each to reach a complete diagnosis. Moreover, bacterial genomic investigation yielded an epidemiological contribution, suggesting an Italian origin of the infection, because the bacteria isolates were more similar to strains endemic in Italian cattle livestock than East European isolates. We emphasize the importance of molecular analyses to trace the origin of infections in terms of both geographical and cross-species tracing. We highlight that paratuberculosis has to be taken into account when dealing with endangered ruminant species, because even the death of a single animal can reduce genetic variability.

**Abstract:**

Paratuberculosis, a chronic disease caused by *Mycobacterium avium* subsp. *paratuberculosis* (MAP), in ten scimitar-horned oryxes (SHOs) hosted in an Italian zoological park and originating from a Slovakian flock, was documented by pathology, molecular, cultural, and serological testing. The infection origin in this threatened species was also investigated by genomic analyses. Following the death of six of the 10 SHOs, serial investigations of dead and alive animals were performed. Necropsy, carried out on five out of six animals, identified intestinal thickening and mesenteric lymphadenomegaly in one of the animals. Histopathology (5/6) revealed lepromatous (2/5) and tuberculoid (2/5) intestinal forms or lack of lesions (1/5). Ziehl-Neelsen and immunohistochemistry stains identified two multibacillary, two paucibacillary forms, and one negative case. MAP was identified by quantitative PCR (qPCR) in tissue samples in five out of five SHOs and was microbiologically isolated from two of the three animals whose fresh tissue samples were available. Fecal samples were collected in four of the six dead animals: all four resulted positive to qPCR and in MAP was isolated in three. ELISA identified MAP-specific antibodies in three of the five dead animals whose serum was available. qPCR identified MAP in the freshly deposited feces of two out of the four alive animals. From the feces of these two animals, MAP was microbiologically isolated in one case. All isolates were classified as MAP type C and profiled as INMV2 and MVS27 by molecular analysis. Genomic analysis of a field isolate revealed clusterization with a European clade but was more similar to Italian than East European isolates. Our findings underline that paratuberculosis should always be considered in zoological parks in which endangered species are hosted. Infection can be subclinical, and multiple combined testing techniques may be necessary.

## 1. Introduction

Paratuberculosis is a chronic granulomatous infectious disease caused by *Mycobacterium avium* subsp. *paratuberculosis* (MAP), an acid-fast (AF) bacterium characterized by long environmental persistence. Ruminants are the most commonly affected species, but several mammals and birds are also susceptible [1,2].

Paratuberculosis is a granulomatous enteritis mostly affecting the ileum and ileocolic junction. Its course can be clinical or subclinical. Persistent diarrhea is a pathognomonic sign in cattle and often leads to animal culling due to progressive and severe emaciation [3]. Small ruminants often exhibit emaciation without diarrhea. The most important transmission route is fecal-oral, but vertical transmission is also reported [4].

Paratuberculosis has a global diffusion, negatively affects animal welfare, and causes conspicuous economic losses due to animal culling, reduced milk production, and testing and therapy expenses [5]. Furthermore, the infection has been recognized as endemic in different wildlife species, making its eradication even more difficult, especially where domestic and wildlife animals share grazing fields [6].

When compared with livestock realities, zoological parks represent a unique context in which diverse animal species are closely housed, meaning that the containment of infectious agents is difficult. Since zoological parks play an important role in the conservation of endangered species, the loss of animals due to infectious diseases can have severe consequences. Consequently, disease outbreaks in zoological parks must be taken into account and carefully monitored. Among the infectious agents reported in zoological parks, MAP has been identified in several ruminant and pseudo-ruminant species [7,8,9,10,11,12,13].

The scimitar-horned oryx (SHO) (*Oryx dammah*) is a Northern Africa native threatened Bovidae included in an international reintroduction program [14]. Only anecdotal information exists regarding paratuberculosis in this species, and no characterization of MAP strains involved in the SHO disease is available [15]. This study described paratuberculosis in an SHO group hosted in a Northern Italy zoological park by pathology, molecular, cultural, and serological testing. Furthermore, the origin of the infection was investigated by molecular epidemiological analyses.

## 2. Materials and Methods

### 2.1. Animals

The study focuses on ten SHOs (numbered from 1 to 10) hosted in a Northern Italy zoological park and derived from a flock imported from Slovakia during 1994–1996.

SHO 1, a 10-year-old male died in 2016, showed diarrhea, weight loss, and submandibular edema.

SHO 2, a severely emaciated 12-year-old female showing severe left carpal joint synovitis unresponsive to treatments, was euthanized 1.5 years after SHO 1 death and submitted for necropsy. Clinical history reported intermittent episodes of profuse diarrhea.

SHO 3 died a few months after SHO 2 with no history of enteric disorders, and SHO 4 was euthanized because of severe emaciation due to profuse persistent diarrhea. Both animals were neutered males, 13.5 (SHO 3) and 4 years old (SHO 4), respectively, and were submitted to necropsy.

SHO 5, a 4.5-year-old neutered male with no history of enteric disorders and SHO 6, a 9.5-year-old neutered male with a clinical history of episodic diarrhea associated with weight loss, both died.

SHOs 7–10—a 7.5-year-old male (SHO 7), and three females (SHOs 8, 9, and 10) aged 4, 3, and 1.5 years—were the remaining live animals of the group.

For the study, no animals were killed nor submitted to stressful procedure. In alive animals, only freshly deposited feces were collected.

### 2.2. Biological Samples

The investigation started after the death of SHO 2, and due to the different status of the animals (dead or alive), different testing and samplings were possible. Details about the matrices analyzed for each SHO are reported in Table 1.

In SHO 1, necropsy was not carried out, and only feces were analyzed.

In SHOs 2–6, necropsies were performed. For SHOs 2–4, specimens of ileocolic junction, mesenteric lymph nodes, and other tissues were collected and fixed in 10% buffered formalin. Before fixation, fresh ileocolic junction and mesenteric lymph node sample were submitted, together with fecal samples, for culture and molecular analyses. Clotted-blood samples were retrieved from heart chambers.

To determine if other animals belonging to the same SHO flock were affected by paratuberculosis, reports and gross pictures of necropsies carried out in the park on two SHOs (SHOs 5 and 6) that died before the study start were retrieved. Moreover, the related formalin-fixed and paraffin-embedded (FFPE) samples were retrieved from the histological archive of the Istituto Zooprofilattico della Lombardia e dell’Emilia Romagna (IZSLER). Furthermore, because post-mortem blood collection is routinely performed by the park veterinary service, frozen sera from SHOs 5 and 6 were also available. In alive SHOs (7–10), freshly deposited feces of each animal were collected. Since blood sampling represents a stressful procedure for SHOs, it was not performed on live animals.

### 2.3. Histology and Immunohistochemistry

Histological samples were routinely processed and paraffin embedded. From each paraffin block, 4 µm thick sections were obtained and stained with hematoxylin and eosin. Further sections from the intestine and mesenteric lymph node of SHOs 2–4 and from the retrospectively retrieved FFPE intestinal specimens (SHOs 5 and 6) were submitted to Ziehl–Neelsen (ZN) staining.

For immunohistochemistry (IHC), 4 µm thick sections of intestine for SHOs 5 and 6, and intestine and mesenteric lymph node for SHOs 2–4 were dewaxed, rehydrated using graded alcohols, and then treated with 3% H_2_O_2_ in ddH_2_O for 30 min to inhibit endogenous peroxidase. Heat-induced antigen retrieval was obtained at 121 °C for 10 min in ddH_2_O in a pressure cooker (Bio SB, Santa Barbara, CA, USA). Then, sections were blocked for 5 min with Novocastra Protein Block (Leica Biosystems, Newcastle Upon Tyne, UK) at room temperature. Polyclonal rabbit anti-MAP antibody (B312, Dako Denmark A/S, Glostrup, DK-2600, DK) was diluted 1:5500 in BOND Primary Antibody Diluent (Leica Biosystems) and incubated overnight at 4 °C. After washing in BOND Wash Solution (Leica Biosystems), the Novolink Max Polymer Detection System (Leica Biosystems) was used. IHC reaction was visualized with the NovaRED Peroxidase Substrate Kit (Vector Laboratories, Burlingame, CA, USA). Sections were counterstained with Mayer’s hematoxylin. Negative controls, obtained by replacing primary antibody with Antibody Diluent, were included in the IHC assay.

### 2.4. IS900-qPCR

For the direct detection of MAP by IS900-quantitative PCR (qPCR), primers, probes, and other PCR conditions were applied as previously described [16]. Notably, after a bead-beating step with acid-washed glass beads (Sigma Aldrich, Milan, Italy) in Tissue Lyser II (Qiagen, Milan, Italy) for 10 min at 30 Hz, DNA extraction from fecal samples and both fresh and FFPE tissue samples were obtained with a DNeasy Blood & Tissue Kit (Qiagen) according to the manufacturer’s instructions. For fecal samples, the bead-beating step was applied before applying a previously described DNA extraction protocol [17]. Briefly, 1–3 g of feces were suspended in 20 mL of sterile distilled water, vortexed, and allowed to settle for 5 min at room temperature. Then, 500 µL of the suspension were collected into tubes containing 200 mg of acid-washed glass beads for the bead-beating step. One hundred and eighty μL of Buffer AL and 20 μL of Proteinase K were added to sterile tubes containing 200 μL of samples. After 10 min of incubation at 70 °C, 210 μL of ethanol were added, and all the lysate obtained was loaded into a QIAamp Column placed in a collected tube. The columns were washed with 500 μL of Buffer AW1 and AW2 and subsequently, the DNA was eluted in 200 μL of elution buffer and used for the qPCR analysis centrifuging the tubes at 16,000× *g* for one min each step.

For the tissue samples, small pieces of tissue containing the mucosa were placed into tubes with glass beads, 180 μL of Buffer ATL, 20 μL of Proteinase K, and 50 μL of 10 mg/mL Lysozyme (Sigma Aldrich, Milan, Italy). After a bead-beading step, the tubes were incubated at 37 °C for 30 min and at 55 °C for 30 min. Afterwards, 200 μL of buffer AL were added, and a 10 min incubation at 70 °C was carried out. The next steps were as described for fecal samples.

Positive samples were classified as strongly positive (less than 28 Cq) or weakly positive (between 28 and 36 Cq) based on the Cq values.

### 2.5. Culture

Culture was performed only for SHOs resulted positive to qPCR and whose fresh samples were available. For MAP isolation, double incubation protocols reported in the paratuberculosis section of the World Organisation for Animal Health (OIE) “Manual of Diagnostic Tests and Vaccines for Terrestrial Animals” and single incubation protocol as described in Savi et al. 2015 were applied to feces and fresh tissue samples, respectively [18,19]. Samples were classified as negative, and weakly (less than 10 colonies per slant), moderately (between 10 and 50 colonies per slant), or strongly (more than 50 colonies per slant) positive [20].

### 2.6. ELISA Test

Serological analyses were carried out using an ELISA commercial test (ID Screen Paratuberculosis Indirect, ID-Vet, Montpellier, France).

### 2.7. Molecular Epidemiological Analyses

MAP isolates were subtyped by specific PCR and for mini- and microsatellite analyses according to a previously described loci scheme [21,22]. MAP isolate from SHO 1 was further analyzed by Whole Genome Analysis (WGA) with a Nextera XT kit (Illumina, Inc., San Diego, CA, USA) and sequencing it on a Miseq platform (Illumina) in a 2 × 250 bp paired-end run. To explore the genomic relationship with other Italian and European MAP isolates, a single nucleotide polymorphism (SNP) analysis was performed with the CFSAN SNP Pipeline using the MAP K-10 genome as reference [23,24]. A SNP-based phylogenic tree was built using the Maximum Likelihood (ML) algorithm in RaxML software (DNASTAR, Inc., Madison, WI, USA) with 100 bootstrap replicates [25]. The genomic analysis included a selection of MAP strains of Type B and S, and all Italian and European isolates of Type C sequenced in previous studies [23].

## 3. Results

### 3.1. Necropsy Findings

SHO 2 was mildly emaciated, with a severe chronic purulent synovitis, and mild hyperemia and flaccidity of distal jejune, ileum, and ileocolic junction. Mesenteric lymph nodes were normal.

SHO 3 showed severe ruminal gaseous distension due to an obstructive foreign body in the reticulo-omasal orifice, which was associated with lung congestion and oedema. Intestine and mesenteric lymph nodes were normal.

SHO 4 was severely emaciated and distal jejune, ileum, and ileocolic junction were severely hyperemic and mildly thickened. Mesenteric lymph nodes were enlarged.

SHO 5 showed flaccidity and severe hyperemia of distal jejunum and ileum. Mesenteric lymph nodes were normal.

SHO 6 showed mild emaciation, moderate pulmonary oedema, right dilatative cardiomyopathy, flaccidity, and severe hyperemia of distal jejune and ileum. Mesenteric lymph nodes were normal.

### 3.2. Histology and Immunohistochemistry

In SHO 2, the intestine was diffusely hyperemic, and lamina propria was infiltrated by numerous epithelioid macrophages admixed with fewer small mature lymphocytes and rare plasma cells (lepromatous form), and, in addition to multifocal mineralization, multifocal macrophage aggregates were visible in the submucosa. In mesenteric lymph nodes, single or aggregated epithelioid macrophages were present. ZN staining revealed numerous AF bacilli within intestinal (multibacillary form) and, to a lesser extent, lymph nodal macrophages. Similarly, MAP antigens were immunohistochemically detected and were numerous within intestinal and scant within lymph nodal macrophages.

SHO 3 showed mild fibrosis of intestinal mucosa, and no lesions were noted in the lymph nodes. Both organs resulted ZN and IHC negative.

**Figure 1:**
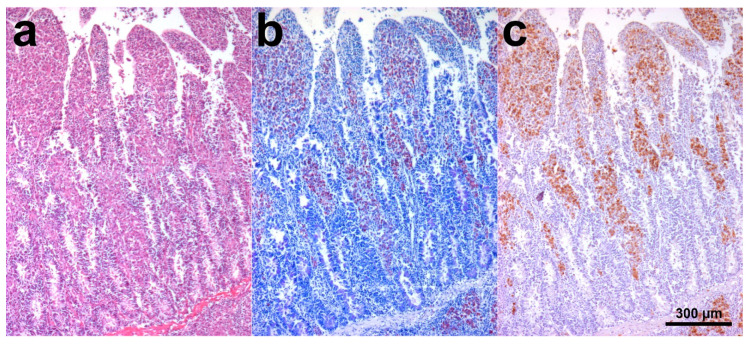
Scimitar-horned oryx (SHO) 4, ileum. (**a**) Numerous epithelioid macrophages were visible in the lamina propria. At the bottom right of the image, a macrophage aggregate is visible in the submucosa. Hematoxylin and eosin (H&E) stain; (**b**) Myriad acid-fast (AF) bacilli were highlighted within intestinal macrophages. Ziehl–Neelsen (ZN) stain; (**c**) *Mycobacterium avium* subsp. *paratuberculosis* (MAP) antigens were abundantly highlighted within intestinal macrophages. Immunohistochemistry (IHC) stain.

**Figure 2:**
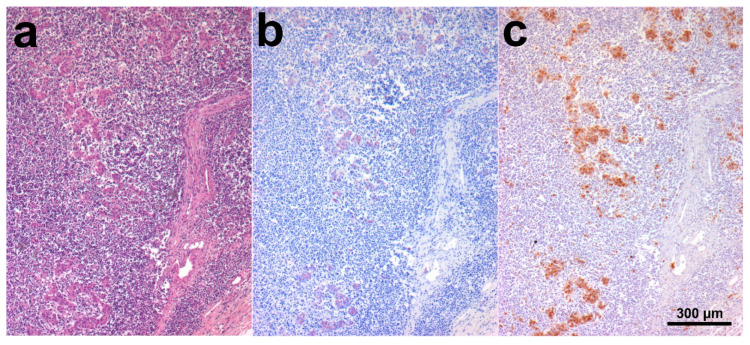
SHO 4, mesenteric lymph node. (**a**) Numerous aggregates of epithelioid macrophages were visible in lymph nodal parenchyma. HE stain; (**b**) A moderate number of AF bacilli were detected within lymph nodal macrophages. ZN stain; (**c**) MAP antigens were moderately highlighted within lymph nodal macrophages. IHC stain.

SHO 4 histology showed dense sheets of epithelioid macrophages (lepromatous form; Figure 1a), admixed with fewer small mature lymphocytes, rare plasma cells, and occasional Langhans giant multinucleated macrophages, in the intestinal lamina propria. Additional intestinal findings were diffuse hyperemia, multifocal mineralization, and macrophage aggregates in the submucosa. Numerous aggregates of epithelioid macrophages were visible in mesenteric lymph nodes (Figure 2a). Myriads of AF bacilli were highlighted within intestinal macrophages (multibacillary form; Figure 1b), while fewer AF bacilli were detected within lymph nodal macrophages (Figure 2b). MAP antigens were abundantly and moderately highlighted, respectively, within intestinal (Figure 1c) and lymph nodal macrophages (Figure 2c).

The SHO 5 intestine was diffusely hyperemic, and lamina propria was infiltrated by numerous small mature lymphocytes and plasma cells, admixed with occasional macrophages (tuberculoid form). One to a few AF bacilli were present within the macrophage cytoplasm (paucibacillary form), in which MAP antigens were also immunohistochemically detected.

The SHO 6 intestine was diffusely hyperemic, and lamina propria was multifocally mineralized and moderately infiltrated by small mature lymphocytes, scattered macrophages (tuberculoid form), and rare plasma cells. One to a few AF bacilli were highlighted in the macrophage cytoplasm (paucibacillary form), in which a positive IHC signal was also detected.

### 3.3. IS900-qPCR

Eight out of ten SHOs resulted positive (Table 1). In particular, the intestines and mesenteric lymph nodes of SHOs 2 and 4 were classified as strongly positive, while the intestines of SHO 3, 5, and 6 were weakly positive. Feces of SHO 1, 2, 4, and 7 were strongly positive, and feces of SHO 3 and 10 were weakly positive. Feces of SHOs 8 and 9 were negative.

### 3.4. Culture

MAP was isolated from 4/6 qPCR positive SHOs whose fresh samples were available. Specifically, MAP was isolated from the tissue and feces of SHOs 2 and 4 were isolated from the feces of SHOs 1 and 7 (Table 1).

### 3.5. ELISA test

SHOs 2, 4, and 5 were positive, whereas SHOs 3 and 6 were negative (Table 1).

### 3.6. Molecular Epidemiological Analyses

All MAP isolates were classified as type C. Mini and microsatellite analyses showed the same INMV2 profile for all isolates (Table S1). According to the MAC-INMV-SSR Database for VNTR analyses, the recovered profile was one of the most diffuse globally (INMV2) [26]. According to a loci scheme already used in Italy (VNTR + SSR), the observed profile was one of the most diffuse among MAP isolates circulating in Italian cattle (MVS27) [22].

**Figure 3:**
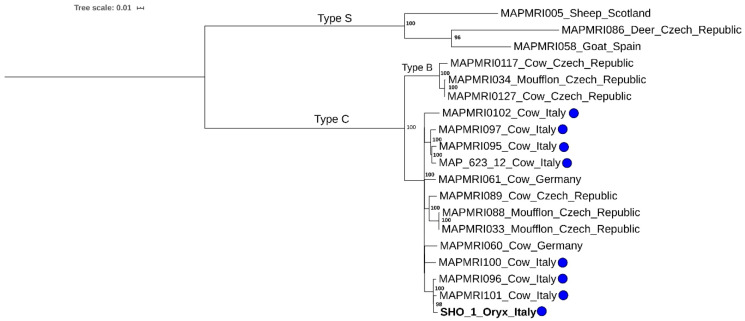
Maximum likelihood phylogenetic tree of MAP isolates selected for this study. Oryx isolate is indicated in bold type, and Italian isolates are indicated with a blue circle. Bootstrap values are reported on the right side of the nodes.

The genomic analysis of a selection of previously sequenced MAP strains highlights that the SHO 1 isolate clusters with Type C isolates, in particular with a monophyletic cluster of Italian isolates (MAPMRI096 and MAPMRI101) (Figure 3) possessing 51 pairwise SNPs with the SHO 1 isolate [23]. This Italian cluster is part of a European clade composed of seven Italian, two German, and three Czech isolates, with maximum pairwise distance to the SHO 1 isolate of 207 SNPs (Figure 3). Raw reads of the isolate from SHO 1 was deposited at EBI under Project number PRJEB38613 (https://www.ncbi.nlm.nih.gov/bioproject/?term=PRJEB38613).

## 4. Discussion

Paratuberculosis occurs globally in both domestic and wild ruminants with important consequences for animal health and welfare. The chronic nature of the disease and the variable progression of infection and immune response often complicate the diagnosis. In addition, different diagnostic techniques often provide different results depending on the disease progression. By applying different diagnostic techniques, we investigated MAP infection in an SHO group, including symptomatic and asymptomatic animals.

Gross findings were poorly specific and were represented by hyperemia and flaccidity of intestinal tracts with only one animal exhibiting mild mucosal thickening and mesenteric lymph nodal enlargement, highlighting that MAP infection should always be considered even when mild and no specific findings are observed.

In two (SHOs 2 and 4) of the five animals submitted to necropsy, histology revealed an abundant intestinal macrophagic infiltrate (lepromatous form), and corresponding lymph nodes were characterized by multifocal macrophagic aggregates. In two other animals (SHOs 5 and 6), moderate to severe lymphoplasmacytic enteritis, hiding rare and scattered macrophages (tuberculoid form), was found [27]. In ruminants, the tuberculoid forms of paratuberculosis are usually visible in the earlier stages of infection. In contrast, the lepromatous forms are found in the later stages [28]. Macrophagic infiltration, the hallmark of mycobacterial infections, can be scant in the tuberculoid forms and hidden by the lymphoplasmacytic component. Consequently, tuberculoid forms are more likely to be missed during histopathological examination, although this is less important from an epidemiological point of view because it is associated with lower environmental MAP shedding than that of lepromatous forms.

In all animals in which tissues were available, with the exception of SHO 3, intestinal tracts (SHOs 2, 4–6) and lymph nodes (SHOs 2 and 4) resulted in positive tests for the presence of AF bacilli and MAP antigens, as detected by ZN and IHC stains, respectively. Both stains allowed classifying two cases (SHOs 2 and 4) as multibacillary and two cases (SHOs 5 and 6) as paucibacillary, also demonstrating the presence of the infection in the tissues harboring scant mycobacteria [29,30]. Therefore, ZN and IHC stains are recommended in all cases in which MAP infection is suspected, even if only mild pathological changes are present. Specifically, ZN staining, which is easy to perform and relatively inexpensive, was found to be a relevant diagnostic tool.

In all dead or euthanized animals (SHOs 1–6), MAP was detected by PCR. This test proved to be effective for both fresh and archival FFPE samples. For SHOs 2–4, there was accordance between results on feces and tissues, which were both positive for MAP. PCR and culture were strongly positive in SHOs 2 and 4 (intestine and mesenteric lymph node) and weakly positive for SHOs 3, 5, and 6 (intestine). However, in SHO 5 and 6, a paucibacillary form was identified, and in SHO 3, the weak positive PCR result could be interpreted as a “pass-through” shedding of MAP because the animal did not show any clinical signs, and all other investigations yielded negative results [31]. As previously reported, this highlights that a positive molecular result is not necessarily associated with pathogen tissue invasion.

Three animals (SHOs 2, 4, and 5) of five (SHOs 2–6) yielded positive results from serological analysis. These results match clinical signs, pathological findings, and PCR results. The two negative SHOs (3 and 6) deserve further comment. SHO 3 showed no clinical signs nor macro-microscopic alterations and was negative for ZN, IHC, and microbiological examination; therefore, the negative serological result supports the hypothesis of SHO3 as a passive-shedder. In contrast, SHO 6, which was positive to ZN and IHC, affected by a paucibacillary form, and serologically negative, could be in the early phase of the disease in which the animal had not yet seroconverted [32].

In alive SHOs (7–10), only feces were tested by PCR and culture. In live animals, PCR on fresh feces is recommended as the test of choice because it is cheap, fast, and highly sensitive compared to culture. Stated this, in the present study, we perform culture only in fecal samples resulted positive at qPCR [33,34].

The isolates circulating in the flock belonged to MAP type C and shared the same mini and microsatellite combinations, suggesting a clonal nature of the infection. The detected profile was previously observed in field isolates from a vast host range of ruminant and non-ruminant wildlife animals and is also the predominant type in Italian bovine specimens [22,26].

Phylogenetic analysis of the SHO 1 MAP isolate revealed that it was more similar to Italian than East European isolates. Consequently, the infection was likely acquired after the original SHO flock was moved from Slovakia and probably from cattle. This last hypothesis is supported by the low number of pairwise SNPs among the SHO 1 isolate and the other Italian members of the monophyletic cluster (MAPMRI096 and MAPMRI101). Specifically, the monophyletic cluster containing the SHO 1 isolate is well supported by the bootstrap resampling, and it is the isolate with the lowest SNP difference among the isolates of all clusters of the Type C clade, suggesting a real genomic relationship of the cluster members.

Finally, considering the age of animals with clinical signs, it must be remarked that all SHOs were adults, four years or older. This finding is more similar to the experience in bovine species than in small ruminants, where signs can occur in younger animals [18,20,31].

## 5. Conclusions

Our results underline the importance of considering paratuberculosis in zoological parks, where endangered species are often hosted. Paratuberculosis could represent a risk for the conservation of rare animals, and it is essential to include it in the panel of diagnostic tests to be performed on hosted animals. We also suggest testing dead animals, in which different diagnostic approaches are combined, with the final aim of fully elucidating the causes of death and defining their health status regarding paratuberculosis.

WGA can help to trace the origin of infections, particularly in the case of moved animals. This study reports the first genome of an MAP strain isolated from SHOs and shows that the strain likely derived from the Italian cattle livestock, in which MAP is endemic.

## Figures and Tables

**Table 1:** Summarized matrices and results of cultural, molecular, and serological tests.

ID	Age (years)	Sex	Samples	qPCR	Culture	ELISA
1	10	M	Feces	Strong positive	Strong positive	
2	12	F	Intestine	Strong positive	Strong positive	
			Mesenteric lymph node	Strong positive	Moderate positive	
			Feces	Strong positive	Weak positive	
			Serum			Positive
3	13.5	M	Intestine	Weak positive	Negative	
			Mesenteric lymph node	NP	NP	
			Feces	Weak positive	Negative	
			Serum			Negative
4	5	M	Intestine	Strong positive	Strong positive	
			Mesenteric lymph node 1	Strong positive	Strong positive	
			Mesenteric lymph node 2	Strong positive	Weak positive	
			Feces	Strong positive	Strong positive	
			Serum			Positive
5	4.5	M	Intestine (FFPE)	Weak positive	NP	
			Serum			Positive
6	10	M	Intestine (FFPE)	Weak positive	NP	
			Serum			Negative
7	7.5	M	Feces	Strong positive	Strong positive	
8	4	F	Feces	Negative	NP	
9	3	F	Feces	Negative	NP	
10	1.5	F	Feces	Weak positive	Negative	

ID: scimitar-horned oryx (SHO) identification, M: male, F: female, NP: not performed, FFPE: formalin-fixed paraffin and embedded samples.

## Supplementary Materials

The following are available online at https://www.mdpi.com/2076-2615/10/11/1949/s1, Table S1: Results of mini- and microsatellite analyses of MAP isolates.
